# Real-world data on treatment and outcomes of patients with primary mediastinal large B-cell lymphoma: a Swedish lymphoma register study

**DOI:** 10.1038/s41408-021-00491-7

**Published:** 2021-05-21

**Authors:** Tove Wästerlid, Sverker Hasselblom, Joel Joelsson, Caroline E. Weibull, Georgios Rassidakis, Birgitta Sander, Karin E. Smedby

**Affiliations:** 1 Department of Medicine Solna, Division of Clinical Epidemiology, Karolinska Institutet and Karolinska University Hospital, Stockholm, Sweden; 2 Department of Hematology, Karolinska University Hospital, Stockholm, Sweden; 3 Department of Research, Development & Education, Region Halland, Halmstad, Sweden; 4 Institute of Medicine, Sahlgrenska Academy at the University of Gothenburg, Gothenburg, Sweden; 5 Department of Oncology-Pathology, Karolinska Institutet, Stockholm, Sweden; 6 Division of Pathology, Department of Laboratory Medicine, Karolinska Institutet and Karolinska University Hospital, Stockholm, Sweden

**Keywords:** Epidemiology, B-cell lymphoma

Dear Editor,

Primary mediastinal large B-cell lymphoma (PMBL) is an aggressive lymphoma subtype that predominantly affects women in their 3rd–4th decade. Due to the rarity of PMBL, optimal treatment, including choice of chemotherapy regimen and necessity of radiotherapy (RT), is not established^1–3^.

In view of the low median age at diagnosis, minimising long-term toxicity while still achieving cure, is essential in ideal PMBL management. To improve knowledge regarding long-term outcomes and optimal management of PMBL we examined patient characteristics, treatment choice, relative survival and excess mortality among all patients registered with PMBL in the population-based Swedish Lymphoma Register (SLR) 2007–2018. Established in 2000, the SLR prospectively records lymphoma-specific patient characteristics and since 2007 also data on active treatment, treatment type and response, with a coverage of ∼95% of all lymphoma cases diagnosed in Sweden. Further, for cases included from the Stockholm area (*n* = 39) central pathology review (by experienced hematopathologists BS and GR) was performed on all patients with available material (*n* = 37) of which all were confirmed to be true PMBL cases. The study was approved by the Regional Board of the Ethical Committee in Stockholm (2015/2028–31/2).

During the study period, 172 patients were registered with a PMBL diagnosis in the SLR, of whom 98 (57%) were women. The median age was 37.5 years (range 18–85). Median follow-up time for patients alive at end of follow-up was 8 years (range 1.7–13.6 years) and 24 (14%) of the patients died. The majority of patients presented with stage I–II disease (*n* = 116, 67%), 154 (90%) had elevated serum-lactate dehydrogenase (S-LDH), and 152 (88%) had a WHO performance status (PS) score of 0–1. Most patients had bulky disease (>10 cm in diameter) at diagnosis (*n* = 119, 69%) and 44 (26%) had extranodal disease (Table 1). For 92% of the patients, PET was used to assess response. Increasing age was associated with poorer survival (HR continuous age = 1.07, 95% CI: 1.05–1.09). Other factors associated with higher mortality rates in unadjusted and age-adjusted Cox models were PS 2–4 (HR = 3.6, 95% CI: 1.4–9.1) and age-adjusted (aa) IPI 2–3 (HR = 3.0, 95% CI: 1.3–6.9).

**Table 1:** Patient characteristics for Swedish PMBL patients diagnosed 2007–2018 overall and stratified by immunochemotherapy regimen and radiotherapy.

	Whole cohort *N* (%)	Immunochemotherapy regimen				Radiotherapy	
		R-CHOEP-14	R-CHOP-14/21	R-Hyper-CVAD	DA-EPOCH-R	Yes	No
*N*	172	90	37	16	11	26	129
Median age (range)	37.5 (18–85)	35 (18–74)	49 (18–83)	38 (21–51)	41 (23–57)	38.5 (20–70)	36 (18–83)
Sex							
Men	74 (43)	38 (42)	18 (49)	5 (31)	5 (45)	15 (58)	51 (40)
Women	98 (57)	52 (58)	19 (51)	11 (69)	6 (55)	11 (42)	78 (60)
WHO Performance status							
0–1	152 (88)	81 (90)	30 (81)	15 (94)	10 (91)	23 (88)	114 (88)
Ann Arbor Stage^a^							
I–II	116 (67)	64 (71)	26 (70)	8 (50)	8 (73)	22 (85)	85 (66)
S-LDH							
Elevated	154 (90)	82 (91)	30 (81)	15 (94)	11 (100)	25 (96)	113 (88)
Extranodal sites							
No	128 (74)	69 (77)	30 (81)	12 (75)	9 (82)	20 (77)	101 (78)
aaIPI							
0–1	111 (64)	61 (68)	24 (65)	9 (56)	7 (64)	20 (77)	82 (64)
2–3	61 (36)	29 (32)	13 (35)	7 (44)	4 (36)	6 (23)	47 (36)
B-symptoms^b^							
Yes	78 (45)	42 (47)	17 (46)	7 (44)	5 (45)	16 (62)	56 (43)
Bulky disease^c^							
Yes	119 (69)	68 (76)	23 (62)	15 (94)	5 (45)	20 (77)	91 (70)
Radiotherapy							
No	129 (83)	74 (82)	32 (86)	14 (88)	8 (72)	0 (0)	129 (100)
Yes	26 (17)	16 (18)	5 (14)	2(12)	3 (27)	26 (100)	0 (0)

Sixteen patients (9%) with missing treatment data. One patient managed with VACOP-B not included in the table to preserve anonymity.

*LDH* lactate Dehydrogenase, *aaIPI* age-adjusted International Prognostic Index.

^a^Three patients had missing data regarding stage.

^b^Eight patients had missing data regarding B-symptoms.

^c^Seven patients had missing data regarding bulky disease.

Treatment information was available for 156 (91%) patients, whom all received immunochemotherapy, apart from one patient who did not receive active treatment. The most commonly administered regimen was R-CHOEP-14 (*n* = 90, 58%). Sixteen patients (10%) received R-Hyper-CVAD (cyclophosphamide, doxorubicin, vincristine, dexamethasone, high dose methotrexate and cytarabine), 37 (24%) R-CHOP-14 (of which 3 were R-CHOP-21), 11 (7%) DA-EPOCH-R and 1 (<1%) R-VACOP-B. The median age was highest among patients treated with R-CHOP (49 years), compared to 35, 38 and 41 years for patients administered R-CHOEP-14, R-Hyper-CVAD or DA-EPOCH-R, respectively. Apart from age, patient characteristics were similar between regimens, but with a slightly higher proportion of patients with PS 2–4 in the R-CHOP group and patients with stage III–IV disease in the R-Hyper-CVAD group. The majority (95%) of patients completed the planned number of cycles, although data on dose intensity was not available. Only 17% (*n* = 26) of all patients with treatment data available, received consolidative RT.

To estimate efficacy and risk for long-term toxicity of the above PMBL treatment, we calculated excess mortality in PMBL patients in a relative survival framework, which captures both the direct and indirect excess mortality associated with the lymphoma. Using the Pohar Perme method, we compared the OS of PMBL patients to the expected survival in a sex-, age- and calendar year-matched cohort from the Swedish general population (assumed lymphoma-free, data obtained via the Human Mortality Database (www.mortality.org)). Follow-up started on the date of diagnosis and ended on the date of death (from any cause) or the 28th of August 2020, whichever came first. For the whole cohort, 2- and 5-year relative survival was 89% (95% CI: 88–99%) and 88% (95% CI: 86–98%). When conditioning on being alive one year after diagnosis, relative survival for the whole cohort was 95% (95% CI: 94–100%). When conditioning on surviving the first two years after diagnosis, excess mortality further diminished (2-year RS 99% (95% CI: 99–100%)) (Fig. 1a–c). This is in line with previous studies that report a normalisation of survival for DLBCL patients with an event-free survival of 24 months^4^, and for patients with Burkitt lymphoma who have not progressed within one year after treatment completion^5^, but has not been shown before for PMBL. This reassures us that the current treatment regimens are efficacious and do not incur fatal long-term toxicity, and also provides important information regarding follow-up routines in the clinics.

**Fig. 1:**
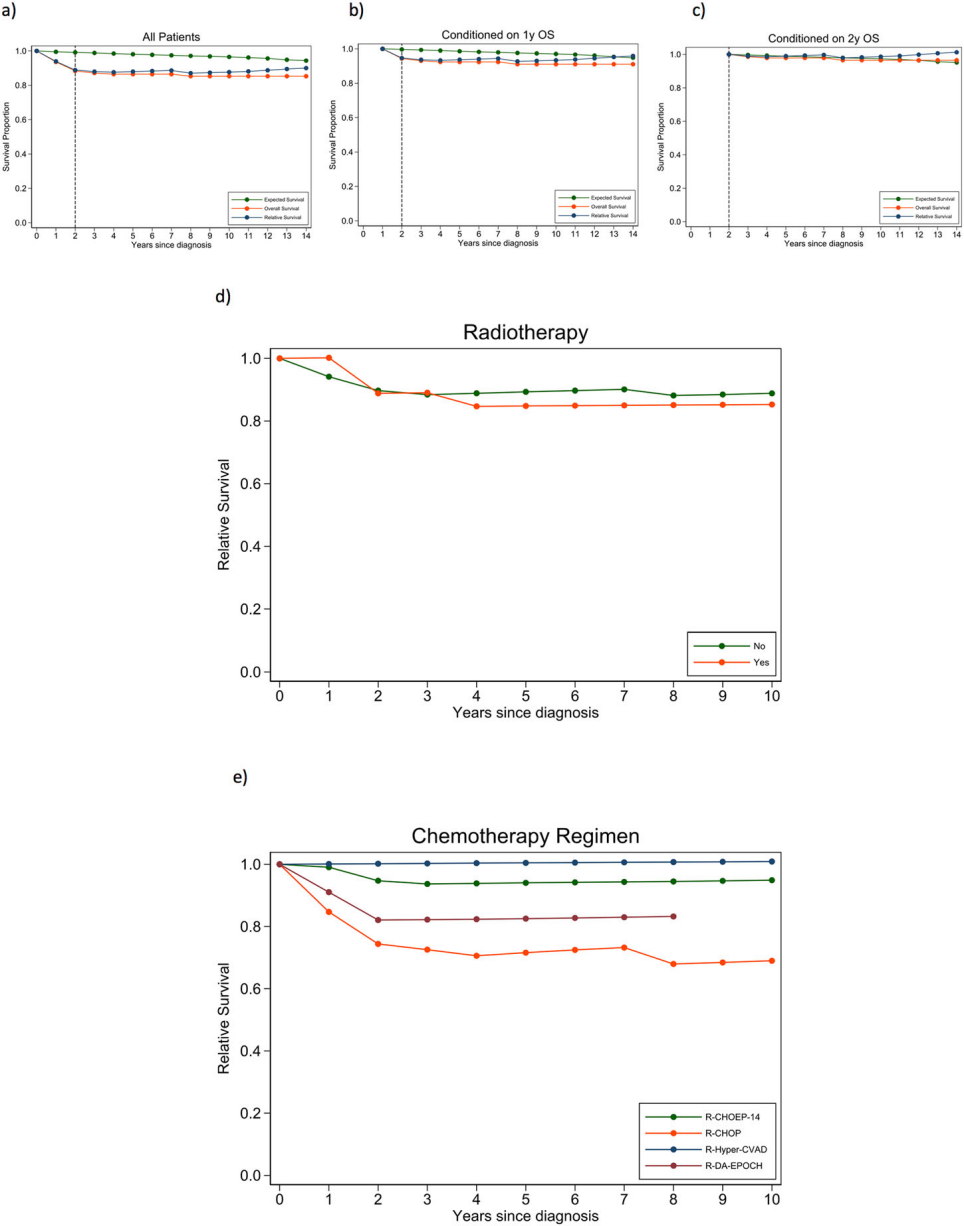
Conditional survival curves followed by relative survival curves by radiotherapy and chemotherapy for Swedish PMBL patients diagnosed 2007-2018. **a** Overall survival, relative survival and expected survival (in the matched general population) among Swedish PMBL patients diagnosed 2007–2018 for all patients. **b** Overall survival, relative survival and expected survival (in the matched general population) among Swedish PMBL patients diagnosed 2007–2018 for patients alive one year after diagnosis. **c** Overall survival, relative survival and expected survival (in the matched general population) among Swedish PMBL patients diagnosed 2007–2018 for patients alive two years after diagnosis. **d** Relative survival stratified by radiotherapy yes/no for Swedish PMBL patients diagnosed 2007–2018. **e** Relative survival stratified by treatment regimen for Swedish PMBL patients diagnosed 2007–2018.

Discussions regarding the optimal management of PMBL have largely focused on more intensive chemotherapy versus R-CHOP + RT, and whether RT can safely be omitted. Only a small proportion (17%) of patients received consolidative RT in our study, and its use was similar between regimens. This limited number precluded robust evaluation of the impact of RT on survival and excess mortality in the present study, although the majority of patients had excellent outcomes without RT^6,7^. Patient characteristics for patients with/without RT were similar apart from a slightly higher proportion of male patients, patients with stage I–II disease, and patients with B-symptoms among those who received RT (Table 1). Outcomes were similar with a two-year RS of 89% (95% CI: 68–96%) for patients who received RT compared to 90% (95% CI: 83–94%) for patients who did not receive RT (Fig. 1d). This is in line with previous studies which show excellent results with various regimens without RT^1^. In addition, in studies where PET-guided RT has been applied, PET-negative patients have safely been managed without RT^3,8^. Further, as false-positive PET scans are common, accumulating evidence suggests that PET-positive patients can be followed with serial PET scans and that biopsy is required to confirm persistent lymphoma, before considering additional therapy^9^.

The limited number of patients in each treatment group, and the inherent confounding by indication in an observational setting, preclude robust conclusions regarding the efficacy of the various treatment regimens in the present study. However, encouraging survival rates were observed especially for R-CHOEP-14 and R-Hyper-CVAD, with 2-year RS rates of 95% (95% CI: 87–98%) and 100%, respectively. Corresponding RS for R-CHOP-14/21 was 74% (95% CI: 56–86%) and 82% (95% CI: 45–95%) with DA-EPOCH-R (Fig. 1e). The R-CHOEP regimen has previously not been specifically studied for PMBL patients, although 87 patients with PMBL were included in the MinT-study, of whom 19 received R-CHOEP^10^. Further, CHOEP has been found to be tolerable among DLBCL patients, and to improve survival compared to CHOP alone among all patients aged below 60 years in the pre-rituximab era^11^. Results from the present study provide support for the continued use of R-CHOEP-14 for PMBL patients. Regarding R-Hyper-CVAD, this regimen has been favoured in certain regions in Sweden in cases with an aggressive clinical presentation, such as in the setting of vena cava superior syndrome. In the current study, this regimen was primarily administered to younger patients with a slightly higher proportion of patients with aaIPI 2–3, compared to the other regimens, with 100% overall survival. Similarly, good results have been demonstrated with other intensive regimens, such as an adapted GMALL B-ALL/NHL 2002 protocol, for PMBL patients^12^. To more justly compare R-Hyper-CVAD with R-CHOEP-14, toxicity data should be taken into consideration, which was unfortunately not available in the present study. Of note, R-Hyper-CVAD also requires in-hospital admission, while R-CHOEP-14 can be administered in an out-patient setting.

The present study provides data on a relatively large number of patients overall, with prospectively recorded clinical data and a regional pathology review, indicating that included patients are true cases of PMBL. Limitations include lack of data regarding detailed PET-response, toxicity, other side-effects of treatment and relapse. Due to the observational design, differences between treatment results should be interpreted cautiously, taking into account treatment selection by both patient and disease characteristics.

To conclude, we report excellent outcomes for PMBL patients, also without RT. Further, we show that the survival of PMBL patients normalises to that of the matched general population among patients who are alive two years after diagnosis, supporting limited disease-oriented follow-up.

## Supplementary information

BCJ check list
